# Th2 cells and macrophages cooperatively induce allergic inflammation through histamine signaling

**DOI:** 10.1371/journal.pone.0248158

**Published:** 2021-03-04

**Authors:** Naruhito Iwasaki, Seigo Terawaki, Kouhei Shimizu, Daisuke Oikawa, Hirokazu Sakamoto, Kishiko Sunami, Fuminori Tokunaga

**Affiliations:** 1 Department of Pathobiochemistry, Graduate School of Medicine, Osaka City University, Osaka, Japan; 2 Department of Otolaryngology-Head and Neck Surgery, Graduate School of Medicine, Osaka City University, Osaka, Japan; University of Michigan Health System, UNITED STATES

## Abstract

Histamine, which is mainly produced by mast cells and basophils, participates in various allergic symptoms, and some studies have reported that macrophages also produce histamine. Moreover, recent studies have revealed that macrophages, especially alternatively activated macrophages (M2) induced by T helper 2 (Th2) cytokines, such as interleukin (IL)-4 and IL-13, participate in the pathogenesis of allergic diseases. The major source of Th2 cytokines is antigen-specific Th2 cells. To elucidate the relationship between histamine, macrophages, and Th2 cells in allergic inflammation, we established a macrophage-Th2 cell co-culture model *in vitro* and an antigen-specific Th2 cell transfer mouse model of rhinitis. *In vitro* analyses indicated that macrophages produce histamine by interacting with antigen-specific Th2 cells through the antigen. Furthermore, Th2 cells and macrophages cooperatively elicited rhinitis in the mouse model. We determined that histamine induces Th2- and macrophage-elicited sneezing responses through H1 receptor signaling, whereas it induces nasal eosinophil infiltrations through H4 receptor signaling. Collectively, these results indicate a novel histamine production mechanism by macrophages, in which Th2 cells and macrophages cooperatively induce nasal allergic inflammation through histamine signaling.

## Introduction

Histamine is a crucial inflammatory mediator produced by many cell types, such as mast cells, basophils, macrophages, and neurons [1]. In patients with allergic rhinitis (AR), the cross-linking of IgE on mast cells/basophils by antigens induces degranulation and histamine release, causing sneezing reactions, nasal discharge, and nasal obstruction [2, 3]. Histamine elicits nasal symptoms by stimulating sensory nerves and acting directly on nasal mucosal blood vessels. Therefore, antihistamine drugs are used as therapeutic drugs for AR worldwide [1, 3]. Four histamine receptors, the H1, H2, H3, and H4 receptors, have been identified, and the antihistamine drugs for AR treatment are targeted to the H1 receptor (H1R) [4]. Antiallergic drug research and development efforts are now targeting the H4 receptor (H4R), since H4R is predominantly expressed on immune cells, such as mast cells, basophils, T cells, and eosinophils [5–7]. However, no anti-H4R drug has been clinically implemented yet.

Macrophages are divided into classically activated macrophages (M1) and alternatively activated macrophages (M2), and play important roles in both innate and acquired immunity [8–10]. M2 macrophages, induced by T helper 2 (Th2) cytokines such as interleukin (IL)-4 and IL-13, reportedly participate in the pathogenesis of allergic diseases [11, 12]. We previously demonstrated that macrophages are involved in nasal allergic reactions in a mouse model [13], and another study also suggested that M2 macrophages contribute to the pathogenesis of chronic rhinosinusitis with nasal polyps [14]. Furthermore, some studies have confirmed that macrophages produce histamine [15–17], a key mediator inducing nasal allergic reactions. Although the histamine derived from macrophages participates in the pathogenesis of atherosclerosis [18], it is not clear whether the histamine from this source is also involved in allergic diseases.

Antigen-specific Th2 cells cause allergic diseases, such as asthma, rhinitis, and atopic dermatitis, by producing Th2 cytokines (IL-4, IL-5, and IL-13). Antigen-specific Th2 cells generally exist as memory Th2 cells *in vivo*, and become activated and mediate allergic inflammation immediately after exposure to specific antigens [19–21]. We previously reported the involvement of antigen-specific Th2 cells in rhinitis, using a mouse model, and found that the nasal activation of antigen-specific Th2 cells induced rhinitis symptoms even in the absence of IgE, mast cells, and basophils [13]. These observations prompted us to investigate the relationship between histamine, macrophages, and Th2 cells in allergic inflammation.

In this study, we identified a novel histamine production mechanism by the interaction of macrophages and Th2 cells, and revealed that Th2 cells and macrophages cooperatively induce nasal allergic inflammation through histamine signaling.

## Materials and methods

### Reagents

Recombinant human IL-2, recombinant mouse IL-4, recombinant mouse IL-12, 4% paraformaldehyde phosphate buffer solution, and diphenhydramine hydrochloride were purchased from FUJIFILM Wako Pure Chemical Corporation (Osaka, Japan). FITC-anti-mouse-CCR3 monoclonal antibodies (mAb) (83101) were purchased from R&D Systems (Minneapolis, MN, USA). Anti-mouse CD3e Ab (145-2C11), anti-mouse CD28 Ab (37.51), APC anti-mouse-CD11b mAb (M1/70), FITC anti-mouse F4/80 mAb (BM8.1), and PE anti-mouse-CD11c mAb (N418) were purchased from Tonbo Biosciences (San Diego, CA, USA). PE-anti-mouse-Siglec-F mAb (E50-2440), APC/Cyanine7 anti-mouse CD45 mAb (30-F11), APC-anti-mouse-F4/80 mAb (BM8), PerCP anti-mouse I-A/I-E mAb (M5/114.15.2), FITC anti-mouse CD44 mAb (IM7), PE anti-mouse CD62L mAb (MEL-14), PE anti-mouse IL-4 mAb (11B11), APC anti-mouse IFN-γ mAb (XMG1.2), and APC-anti-mouse-CD62L mAb (MEL-14) were purchased from Biolegend (San Diego, CA, USA). APC-anti-mouse-DO11.10TCR mAb (KJ1-26) and PerCP-Cy5.5-anti-mouse-CD4 mAb (RM4-5) were purchased from eBiosciences (San Diego, CA, USA). Anti-mouse IL-4 mAb (11B11) and Anti-mouse-IFNγ mAb (R4-6A2) were prepared in our laboratory. Ovalbumin (OVA) (grade V) was purchased from Sigma-Aldrich Japan (Tokyo, Japan). The I-Ab/I-Ad OVA helper peptide (OVApep) (aa 323–339) was purchased from Medical & Biological Laboratories (Nagoya, Japan). Clodronate-containing liposomes were purchased from Hygieia Bioscience (Osaka, Japan). Anti-mouse CD4 magnetic particles (GK1.5), anti-mouse CD11b magnetic particles (M1/70), and APC magnetic particles were purchased from BD Biosciences (San Diego, CA, USA). The potent and selective H4 receptor antagonist, JNJ7777120, was purchased from Cayman Chemical (Ann Arbor, MI, USA).

### Mice

BALB/c wild-type (WT) mice and BALB/c-background DO.11.10^+^ mice were maintained at the Osaka City University animal facilities. BALB/c-background histidine decarboxylase-deficient (*Hdc*^*-/-*^) mice were provided by Dr. Koubun Yasuda (Department of Immunology, Hyogo College of Medicine). BALB/c-background DO.11.10^+^ mice were backcrossed for more than 12 generations. BALB/c-background *Hdc*^-/-^ mice were backcrossed for at least 10 generations. These mice were maintained under specific pathogen-free conditions. All mouse experiments were performed with the approval of the Institutional Animal Care Committee of Osaka City University, in accordance with their guidelines (No. 626, No. 629, and No. 16034).

### Preparation of bone marrow-derived macrophages (BMDMs) and splenic macrophages

BMDMs were generated from bone marrow precursor cells in RPMI 1640, supplemented with 10% fetal bovine serum, 50 μM 2-mercaptoethanol, 1 mM sodium pyruvate, penicillin and streptomycin, and 5% L929 cell culture supernatant. Bone marrow precursor cells were cultured in 24-well plates at 5 × 10^5^ cells/1 ml medium/well for 7 days, and the same medium was replenished every 2 or 3 days. To isolate splenic macrophages, spleens were dissected from WT or *HDC*^-/-^ mice and single-cell suspensions were prepared by sieving and gentle pipetting. Splenic macrophages (F4/80^+^CD11b^+^) were isolated by a BD IMagTM cell separation system, using anti-mouse CD11b magnetic particles, APC-anti-mouse-F4/80 mAb, and APC magnetic particles. The purity of splenic macrophages (frequencies of F4/80^+^CD11b^+^ cells in total isolation cells) was 99.5% (S1 Fig).

### *In vitro* Th cell differentiation and re-stimulation of Th cells

For DO11.10^+^ Th2 differentiation, spleens were dissected from naive DO11.10^+^ mice and single-cell suspensions were prepared by sieving and gentle pipetting. Naive CD4^+^ T cells (CD4^+^CD62L^+^) were isolated by a BD IMagTM cell separation system using anti-mouse CD4 magnetic particles, APC-anti-mouse-CD62L mAb, and APC magnetic particles. DO11.10^+^CD4^+^CD62L^+^ T cells were cultured in 6-well plates at 3 × 10^5^ cells/3 ml/well with 100 pM IL-2, 20 ng/ml IL-4, 10 μg/ml anti-IFN-γ mAb, and 1 μM OVApep, in the presence of 3 × 10^6^ irradiated conventional antigen presenting cells (APCs) from BALB/c splenocytes, cultured in complete medium [RPMI 1640 medium supplemented with 10% fetal bovine serum, 50 μM 2-mercaptoethanol, 1 mM sodium pyruvate, penicillin and streptomycin]. For Th1 cells differentiation, DO11.10^+^CD4^+^CD62L^+^ T cells were cultured in 6-well plates at 3 × 10^5^ cells/3 ml/well with IL-2 (100 pM), IL-12 (20 ng/ml), anti-IL-4 mAb (40 μg/ml), and OVA peptide (323–339) (1 μM) in the presence of 3 × 10^6^ irradiated conventional antigen presenting cells (BALB/c splenocytes) in complete medium. After 5 days, the cells were collected and washed. Th2- or Th1-polarlized cells were re-stimulated with anti-CD3 mAb (2 μg/ml for coating) and anti-CD28 mAb (2 μg/ml) at 1×10^6^ cells/0.5ml complete medium/well in 24-well plates. 24 hours later, cells were collected for flow cytometry analysis. We confirmed the differentiation of Th2 cells (IL-4 positive and IFN-γ negative) and Th1 cells (IL-4 negative and IFN-γ positive) by intracellular staining (S2 Fig).

### *In vitro* co-culture model

BMDMs (5 × 10^5^ cells) or splenic macrophages were co-cultured in 24-well plates in 0.5 ml complete medium/well with naïve CD4 T cells or *in vitro* differentiated OVA-specific Th2 cells or Th1 cells from DO11.10^+^CD4^+^ T cells or *Hdc*^-/-^DO11.10^+^CD4^+^ T cells (1× 10^6^ cells/well), in the presence or absence of 1 μM OVApep. After 24 h, the culture supernatants were collected for histamine measurement.

### Mouse model

For the Th2 cell transfer model, mice were intravenously injected with OVA-specific Th2 cells (5–9 × 10^6^ cells/mouse), and then left untreated for 5 weeks. Mice were intranasally (i.n.) administered 20 μl of PBS or 50 mg/ml OVA for 4 consecutive days (from day 1 to 4). To deplete macrophages, mice were intraperitoneally (i.p.) injected with clodronate liposomes (100 μl/mouse) on day 3, six hours after the nasal challenge. For H1R treatment, mice were intraperitoneally injected with diphenhydramine (500 μg/400 μl PBS/mouse), one day before the first OVA challenge and 60 min before the intranasal challenge with OVA. For the H4R treatment, mice were intraperitoneally injected with JNJ7777120 (500 μg/400 μl PBS/mouse), one day before the first OVA challenge and 60 min before the intranasal challenge with OVA. Immediately after each intranasal challenge, the frequency of sneezing was counted for 10 min. Mice were sacrificed 24 h after the final nasal challenge, and the noses were dissected to analyze the infiltrating inflammatory cells. For the macrophage-depleted experiment, mice were sacrificed just after the final nasal challenge, and the noses were dissected to analyze the infiltrating inflammatory cells.

### Mouse sneezing analysis

Awake mice were intranasally administered 20 μl of challenge reagents. Immediately after the challenge, each mouse was placed in a standard breeding cage without bedding (one mouse per cage). An investigator directly monitored the mouse behavior, and the sneezing behavior was counted for 10 min.

### Quantitative PCR analysis

Total RNAs from BMDMs were isolated using RNeasy MiniKit (Qiagen, Venlo, Netherlands) and cDNA was synthesized using ReverTra Ace (Toyobo, Osaka, Japan). The expression of genes was quantified with Power SYBR Green PCR Master Mix (Applied Biosystems) and 7500 FAST Real Time PCR system (Applied Biosystems) by the standard curve method, according to the manufacturers’ instructions, using the following oligonucleotides: *Arg 1* sense, 5’-CATTGGCTTGCGAGACGTA-3’; *Arg 1* anti-sense, 5’-ATCACCTTGCCAATCCCCAG-3’; *Retnla* sense, 5’-TACTTG CAACTGCCTGTGCT-3’; *Retnla* anti-sense, 5’- TCAAAGCTGGGTTCTCCACC-3’; *18S rRNA* sense, 5’-GATGCCCTTAGATGTCCGGG-3’; *18S rRNA* anti-sense, 5’-ATGGGGTTCAACGGGTTACC-3’ The results were shown as the relative expression normalized to the expression of a gene encoding eukaryotic 18S rRNA.

### Histamine measurement

Histamine was measured with an EIA kit (Bertin Pharma, Montigny-le-Bretonneux, France) according to the manufacturer’s instructions.

### Flow cytometry analysis

Noses were minced with scissors and digested with 150 U/ml collagenase and 10 μg/ml DNase I for 50 min at 37°C. Spleens were homogenized. After filtration with a cell strainer, the red blood cells from the cell suspension were lysed. Cells were incubated with antibodies against CCR3, CD4, CD45, DO11.10TCR, Siglec-F, F4/80, CD11c, CD11b, and MHC class Ⅱ on ice for 30 min, and then washed twice with PBS. Stained cells were analyzed by a FACS LSRⅡ flow cytometer (BD Biosciences) and the FlowJo software (version 10, Tree Star Inc., Ashland, OR, USA). For nasal analysis, singlet CD45^+^ cells were gated as nasal hematopoietic cells. CD45^+^CD4^+^DO11.10TCR^+^ cells and CD45^+^CCR3^high^Siglec-F^high^ cells were defined as OVA-specific DO11.10^+^ Th2 cells and eosinophils, respectively. The data are shown as frequency (% in nasal CD45^+^ cells). For splenic analysis, F4/80^+^CD11b^int^ cells and CD11c^+^MHC-Ⅱ^high^ cells were defined as macrophages and DCs, respectively. The data are shown as frequency (% in total cells). For intracellular staining, Th2 cells and Th1 cells were incubated with anti-CD3/CD28 Abs for 24h. Cytokine productions (IL-4 and IFN-γ) were measured using Foxp3 / Transcription Factor Staining Buffer Set (eBiosciences, San Diego, CA, USA), according to the manufacturer’s instructions.

### Histology

After stripping the facial skin, the mouse noses were demerged between the upper and lower jaws, and noses were removed. Samples were fixed in 4% paraformaldehyde phosphate buffer solution at 4°C for 1 days and decalcified in 0.125 M EDTA solution for 7 days at 4°C. The EDTA solution was changed 2days later. After decalcification, tissues were embedded in paraffin, cut into 4 μm coronal sections, and stained with hematoxylin and eosin (HE).

### Statistics

The two-tailed Student’s t-test and one-way ANOVA followed by Bonferroni’s test were used to determine the statistical significance between two groups and among more than two groups, respectively. Two-way ANOVA followed by Bonferroni’s test was used for analyzing the statistics of time-course experiments and the influence of two different categories. P values less than 0.05 were considered statistically significant.

## Results

### BMDMs produce histamine by interacting with antigen-specific Th2 cells through the antigen

**Fig 1:**
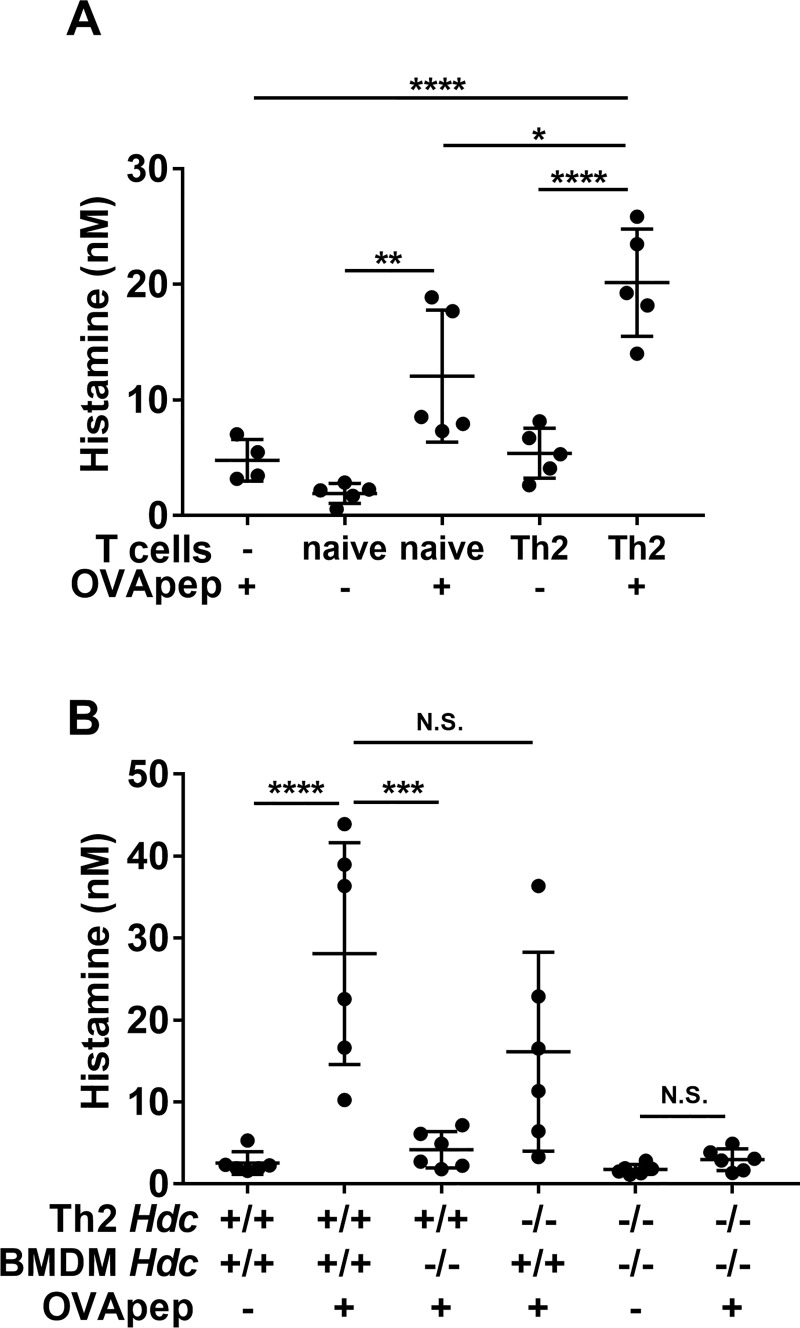
Macrophages produce histamine by interacting with antigen-specific Th2 cells. (**A** and **B**) BMDMs and naïve CD4 T cells or OVA-Th2 cells were co-cultured in the presence or absence of OVApep for 24 h, and then histamine production was measured in the culture supernatants. (**A**) Co-culture of WT-BMDMs and naïve CD4 T cells or WT-OVA-Th2 cells. (**B**) Co-culture of WT or *Hdc*^-/-^BMDMs with WT or *Hdc*^-/-^OVA-Th2 cells. Pooled data from 5 (**A**) or 2 (**B**) independent experiments are shown by means±S.D., n = 4–5 (**A**), n = 6 (**B**). *P<0.05, **P<0.01, ***P<0.001, ****P<0.0001, N.S. not significant.

We first investigated the relationship between histamine, macrophages, and Th2 cells *in vitro*. We initially hypothesized that macrophages could produce histamine by interacting with antigen-specific Th2 cells through the antigen, since macrophages generally function as APCs *in vivo* [8, 9]. To confirm this hypothesis, we established a macrophage-Th2 cell co-culture model *in vitro*. BMDMs were obtained from WT mice, and DO11.10^+^ mouse-derived naïve CD4^+^ T cells, which express OVA (aa 323–339)-specific T cell receptors, were differentiated into Th2 cells (OVA-Th2 cells) *in vitro*. We co-cultured BMDMs and naïve CD4^+^ T cells or OVA-Th2 cells for 24 h with or without OVApep, and measured the histamine concentration in the culture supernatant. In the presence of OVApep, the culture supernatants of BMDMs and naïve CD4 T cells or OVA-Th2 cells contained higher amounts of histamine than those in the absence of OVApep (Fig 1A). Moreover, the amount of histamine produced from the co-culture of BMDMs and Th2 cells with OVApep was significantly increased, compared to that of BMDMs and naïve CD4 T cells with OVApep (Fig 1A). We also confirmed that OVApep alone did not induce histamine production from macrophages (Fig 1A) and investigated the expression of M2 macrophage markers, *Arg 1* and *Retnla* (also known as *Fizz1*), in BMDMs co-cultured with Th2 cells or naïve T cells, by real time PCR. BMDMs co-cultured with Th2 cells in the presence of OVApep showed significantly increased mRNA expression levels of *Arg 1* and *Retnla*, compared to those of BMDMs alone or BMDMs co-cultured with naïve CD4 T cells in the presence of OVApep (S3 Fig). These data demonstrated that the co-culture of BMDMs and Th2 cells with antigen could produce numerous histamine.

We next investigated whether macrophages were the histamine source in this co-culture model, because a previous study revealed that T cells can produce histamine [22]. Therefore, we prepared BMDMs from WT and histidine decarboxylase-deficient *Hdc*^-/-^ mice [23], which cannot synthesize histamine. In addition, to evaluate the contribution of histamine from Th2 cells, we prepared *Hdc*^-/-^DO11.10^+^ mice. *Hdc*^-/-^DO11.10^+^ mouse-derived naïve CD4 T cells were *in vitro* differentiated into Th2 cells that could not produce histamine (*Hdc*^-/-^OVA-Th2 cells). We then co-cultured WT or *Hdc*^-/-^BMDMs and WT or *Hdc*^-/-^OVA-Th2 cells for 24 h with or without OVApep, and measured the histamine concentrations in the culture supernatants. As shown in Fig 1B, the amount of histamine produced from the co-culture of *Hdc*^-/-^BMDMs and WT-OVA-Th2 cells with OVApep was markedly decreased, as compared to that of WT-BMDMs and WT-OVA-Th2 cells with OVApep. On the other hand, the amount of histamine produced from the co-culture of WT-BMDMs and *Hdc*^-/-^OVA-Th2 cells with OVApep was comparable to that of WT-BMDMs and WT-OVA-Th2 cells with OVApep. These results indicated that macrophages could produce histamine by interacting with antigen-specific Th2 cells through the antigen. Furthermore, we also confirmed that BMDMs could produce histamine by interacting with antigen-specific Th1 cells through the antigen, in the same manner to the co-culture of BMDMs and OVA-Th2 cells (S4 Fig).

### Splenic macrophages produce histamine by interacting with antigen-specific Th2 cells through the antigen

**Fig 2:**
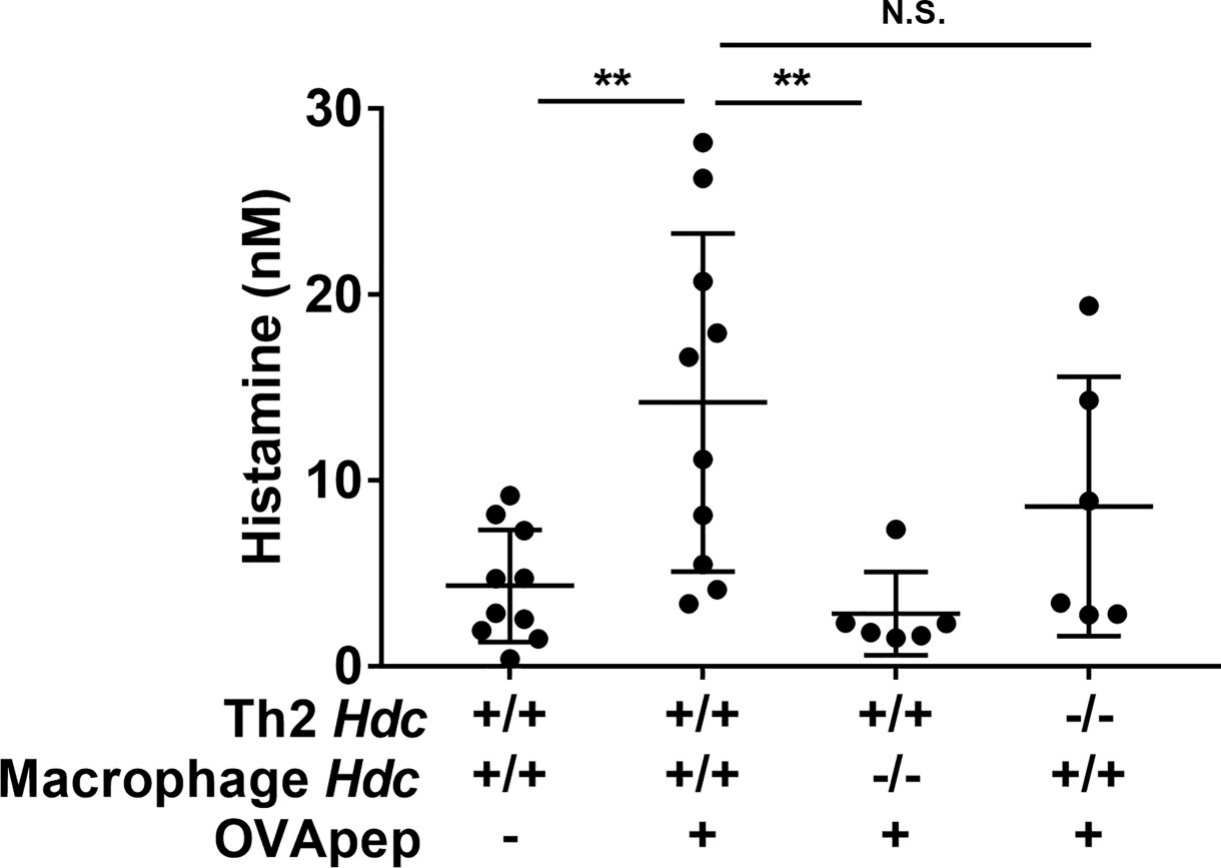
Splenic macrophages produce histamine by interacting with antigen-specific Th2 cells through the antigen. Splenic macrophages (WT or *Hdc*^-/-^macrophages) and OVA-Th2 cells (WT or *Hdc*^-/-^OVA-Th2 cells) were co-cultured in the presence or absence of OVApep for 24 h, and then histamine production was measured in the culture supernatants. Pooled data from 3 independent experiments are shown by means±S.D., n = 6–10. **P<0.01, N.S. not significant.

We next investigated whether *in vivo* macrophages could produce histamine in a similar manner to BMDMs. We isolated splenic macrophages (F4/80^+^CD11b^+^ cells) from WT or *Hdc*^-/-^ mice by a BD IMagTM cell separation system. We then co-cultured WT or *Hdc*^-/-^ splenic macrophages and WT or *Hdc*^-/-^OVA-Th2 cells for 24 h with or without OVApep, and measured the histamine concentrations in the culture supernatants. In the presence of OVApep, the culture supernatant of WT-splenic macrophages and OVA-Th2 cells contained a significantly higher amount of histamine than that without OVApep (Fig 2). In addition, the amount of histamine produced from the co-culture of *Hdc*^-/-^ splenic macrophages and WT-OVA-Th2 cells with OVApep was significantly decreased, as compared to that of WT-splenic macrophages and WT-OVA-Th2 cells with OVApep (Fig 2). On the other hand, the amount of histamine produced from the co-culture of WT-splenic macrophages and *Hdc*^-/-^OVA-Th2 cells with OVApep was comparable to that of WT-splenic macrophages and WT-OVA-Th2 cells with OVApep (Fig 2). These data were totally consistent with the observations of BMDMs. Taken together, we revealed that splenic macrophages could also produce histamine by interacting with antigen-specific Th2 cells through the antigen. Furthermore, we also confirmed that splenic macrophages could not produce histamine by interacting with antigen-specific Th1 cells through the antigen, in the same manner to the co-culture of splenic macrophages and OVA-Th2 cells (S5 Fig).

### Th2 cells and macrophages elicit rhinitis

**Fig 3:**
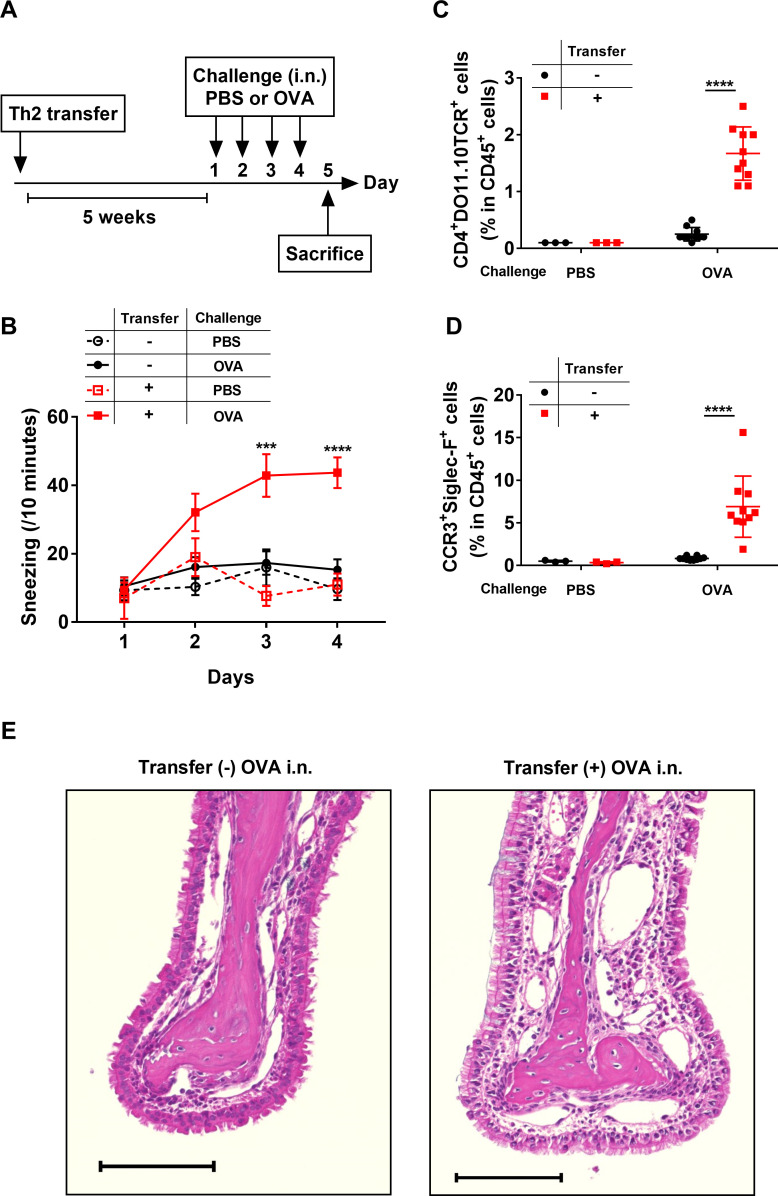
Th2 cells elicit rhinitis. (**A**) Experimental schema. WT mice were adoptively transferred with OVA-Th2 cells, and maintained for 5 weeks. Mice were then intranasally challenged with PBS or OVA. (**B**) Number of sneezes was counted for 10 min immediately after each intranasal challenge. (**C**, **D**) At 24 h after the final challenge, the frequencies of OVA-Th2 cells (CD4^+^DO11.10TCR^+^ cells in CD45^+^ cells) (**C**) and eosinophils (CCR3^+^Siglec-F^+^ cells in CD45^+^ cells) (**D**) in nasal mucosa were examined by FACS. (**E**) HE staining of nasal mucosa. Bar = 100 μm. Pooled data from 3 independent experiments are shown by means±SEM (**B**) or means±S.D. (**C** and **D**), n = 3 (PBS group), n = 10 (OVA group). ***P<0.001, ****P<0.0001. In (**B**), the transfer+ and OVA challenge group was compared with the transfer- and OVA challenge group.

In this study, we aimed to elucidate the mechanisms of allergic rhinitis. Thus, we next investigated the relationship between histamine, macrophages, and Th2 cells *in vivo*, using a mouse model of rhinitis. Th2 cells generally exist as memory Th2 cells *in vivo*; therefore, in this study, we established an OVA-specific Th2 cell transfer model to mimic the physiological conditions *in vivo*. The mice that received OVA-Th2 cells were maintained for 5 weeks to generate memory OVA-Th2 cells, and then challenged with intranasal OVA for 4 consecutive days (days 1–4) (Fig 3A). We first examined the frequencies of OVA-Th2 cells (CD4^+^DO11.10TCR^+^ cells in CD45^+^ cells) in spleen (secondary lymphoid tissue), 5 weeks after transfer, without intranasal OVA challenge, by flow cytometry. We confirmed that OVA-Th2 cells existed in spleen (S6 Fig). These data indicated that OVA-Th2 cells homed to secondary lymphoid tissues within 5 weeks. Furthermore, we investigated whether OVA-Th2 cells differentiated into memory T-like phenotype. Central memory (CD44^+^CD62L^+^) T cells, which are one of the memory T cells, home to T cell areas of secondary lymphoid tissues [24]. Thus, we compared splenic CD44^+^CD62L^+^ OVA-Th2 cells left for 5 weeks after transfer (5 weeks-Th2 cells) with those left for 2 days after transfer (2 days-Th2 cells). The frequencies of central memory (CD44^+^CD62L^+^) T cells were significantly increased in 5 weeks-Th2 cells, compared to those in 2 days-Th2 cells (S6 Fig). These data suggested that OVA-Th2 cells differentiated into memory T-like phenotype within 5 weeks.

The OVA challenge elicited sneezing in Th2-transferred mice, but not non-transferred mice (Fig 3B). Furthermore, we analyzed the nasal infiltrations of Th2 cells and eosinophils by flow cytometry, and confirmed that CD4^+^DO11.10TCR^+^ Th2 cells and CCR3^+^Siglec-F^+^ eosinophils accumulated in the noses of Th2-transferred mice, but not non-transferred mice, on day 5 (Fig 3C and 3D, S6 Fig). Moreover, histologic analysis revealed that mice transferred with Th2 cells and challenged with OVA showed increased infiltrations of inflammatory cells and multilayer formation of the epithelium in the nasal mucosa, compared to mice challenged with OVA without Th2 transfer (Fig 3E). These results indicated that the activation of nasal Th2 cells is essential to induce the sneezing responses.

**Fig 4:**
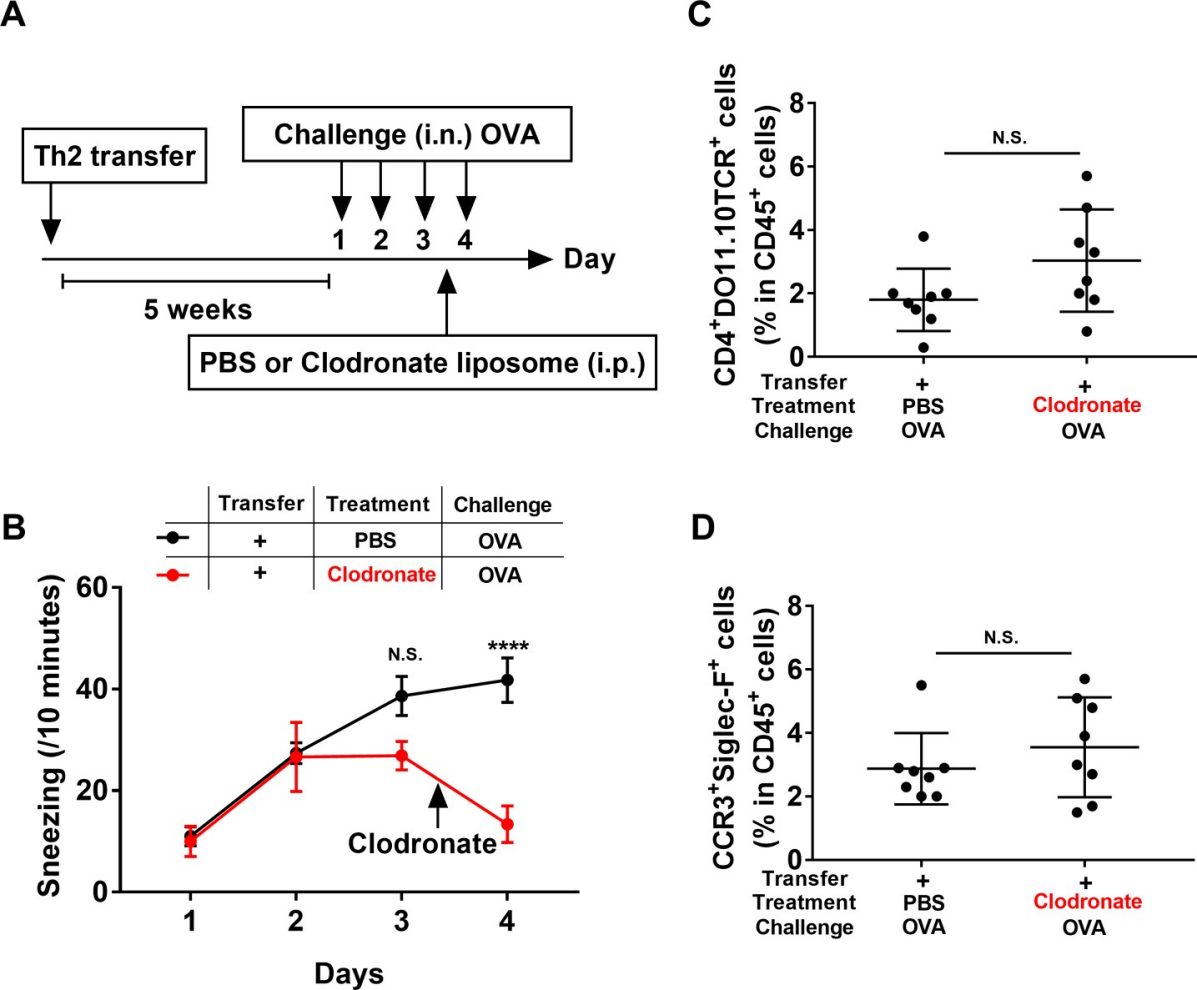
Macrophages are essential for Th2-elicited sneezing responses. (**A**) Experimental schema. WT mice were adoptively transferred with OVA-Th2 cells. After 5 weeks, mice were intranasally challenged with OVA. On day 3, 6 h after the third nasal challenge, mice were intraperitoneally injected with PBS or clodronate-containing liposomes. (**B**) Number of sneezes was counted for 10 min immediately after each intranasal challenge. (**C**, **D**) Immediately after the final challenge, the frequencies of OVA-Th2 cells (CD4^+^DO11.10TCR^+^ cells in CD45^+^ cells) (**C**) and eosinophils (CCR3^+^Siglec-F^+^ cells in CD45^+^ cells) (**D**) in nasal mucosa were examined by FACS. Pooled data from 2 independent experiments are shown by means±SEM (**B**) or means±S.D. (**C** and **D**), n = 8. ****P<0.0001, N.S. not significant.

To further investigate whether macrophages were involved in the rhinitis elicited by the Th2 cell transfer and OVA challenge, macrophages were depleted by an intraperitoneal injection of clodronate-containing liposomes on day 3, 6 h after the third OVA challenge (Fig 4A). We confirmed macrophage depletion by flow cytometry. The clodronate treatment depleted the F4/80^+^CD11b^int^ macrophages, but did not affected the CD11c^+^MHC-Ⅱ^+^ dendritic cells (DC) in mouse spleens (S7 Fig). Macrophage depletion significantly decreased the Th2-elicited sneezing on day 4 (Fig 4B), but did not affect the nasal infiltrations of Th2 cells and eosinophils (Fig 4C and 4D). These data suggested that macrophages are involved in Th2-elicited sneezing responses, but not in the activation of nasal Th2 cells.

### Histamine is involved in Th2- and macrophage-elicited rhinitis

**Fig 5:**
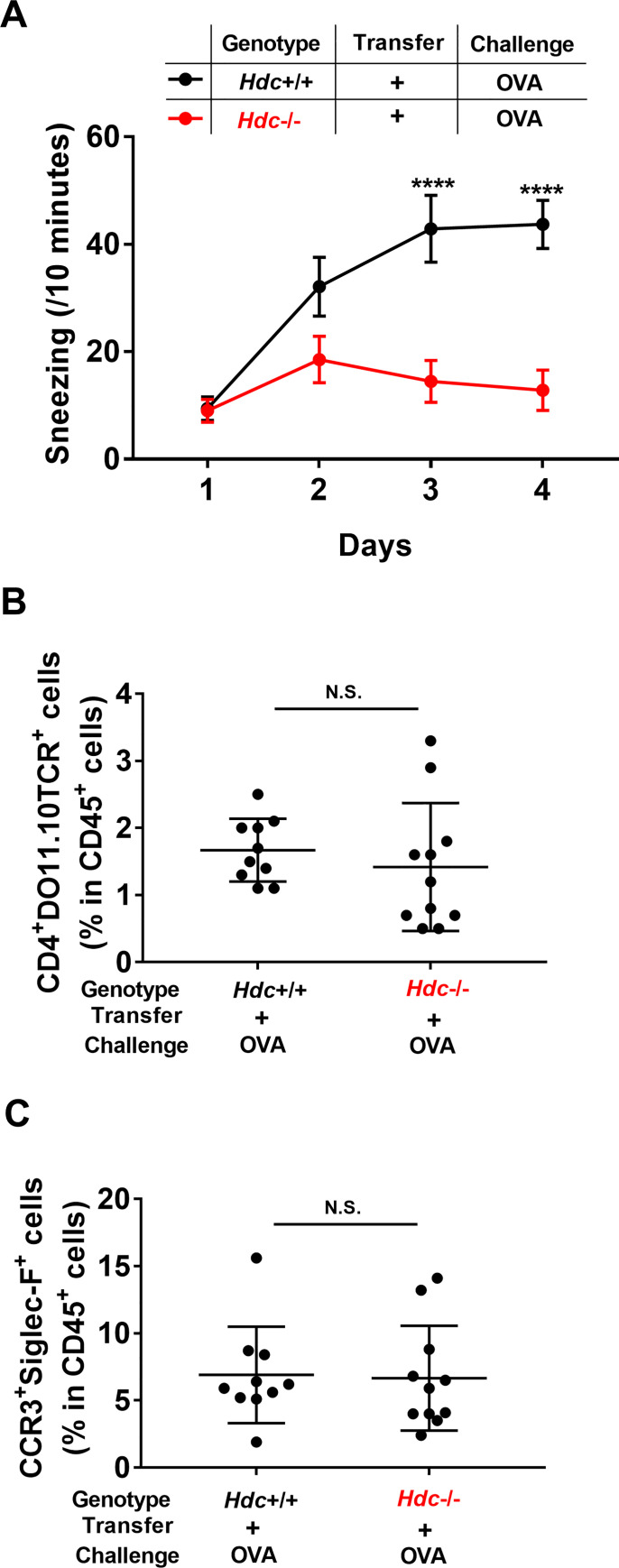
Histamine is essential for Th2- and macrophage-elicited sneezing. WT *(Hdc*^+/+^) or *Hdc*^-/-^ mice were adoptively transferred with OVA-Th2 cells. After 5 weeks, mice were intranasally challenged with OVA. (**A**) Number of sneezes was counted for 10 min immediately after each intranasal challenge. (**B**, **C**) At 24 h after the final challenge, the frequencies of OVA-Th2 cells (CD4^+^DO11.10TCR^+^ cells in CD45^+^ cells) (**B**) and eosinophils (CCR3^+^Siglec-F^+^ cells in CD45^+^ cells) (**C**) in nasal mucosa were examined by FACS. Pooled data from 2 independent experiments are shown by means±SEM (**A**) or means±S.D. (**B** and **C**), n = 10–11. ****P<0.0001, N.S. not significant.

We next investigated whether histamine was involved in the Th2- and macrophage-elicited rhinitis. *Hdc*^-/-^ mice were transferred with OVA-Th2 cells, and then challenged with intranasal OVA 5 weeks later. The OVA challenge failed to elicit sneezing in Th2-transferred *Hdc*^-/-^ mice (Fig 5A). However, the nasal infiltrations of Th2 cells and eosinophils in *Hdc*^-/-^ mice were comparable to those in WT mice (Fig 5B and 5C). Taken together, these observations suggested that the Th2- and macrophage-elicited sneezing responses are mediated by histamine.

### Histamine induces Th2- and macrophage-elicited sneezing responses through H1R signaling and nasal eosinophil infiltrations through H4R signaling

**Fig 6:**
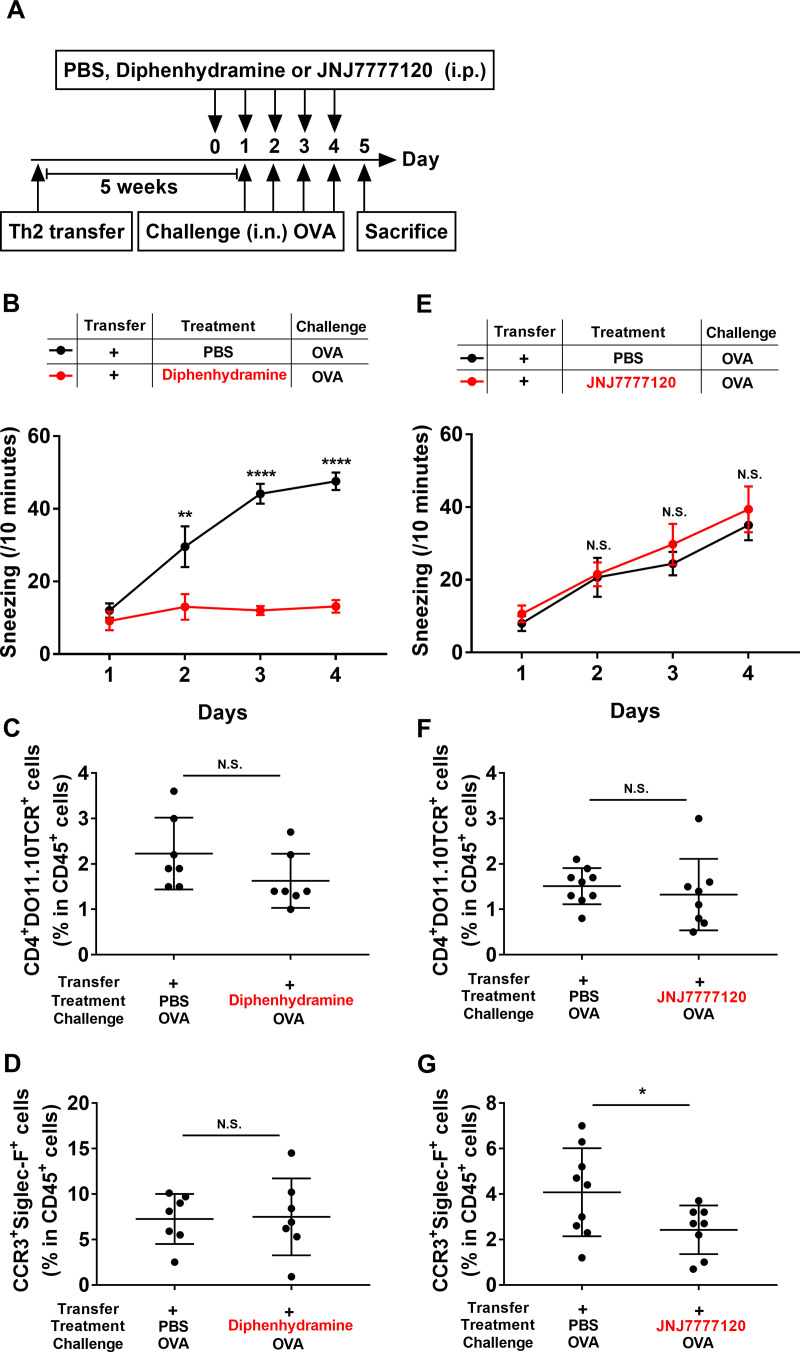
Antagonists against H1R and H4R are effective for Th2- and macrophage-elicited rhinitis. (**A**) Experimental schema. WT mice were adoptively transferred with OVA-Th2 cells. After 5 weeks, the mice were intranasally challenged with OVA. Mice were intraperitoneally injected with PBS, diphenhydramine (500 μg) or JNJ7777120 (500 μg), one day before the first OVA challenge and 60 min before each subsequent OVA challenge. (**B** and **E**) Number of sneezes was counted for 10 min immediately after each intranasal challenge. (**C**-**G**) At 24 h after the final challenge, the frequencies of OVA-Th2 cells (CD4^+^DO11.10TCR^+^ cells in CD45^+^ cells) (**C** and **F**) and eosinophils (CCR3^+^Siglec-F^+^ cells in CD45^+^ cells) (**D** and **G**) in nasal mucosa were examined by FACS. Pooled data from 2 independent experiments are shown by means±SEM (**B** and **E**) or means±S.D. (**C**, **D**, **F** and **G**), n = 7–9. *P<0.05, **P<0.01, ****P<0.0001, N.S. not significant.

Having determined that histamine is involved in the Th2- and macrophage-elicited sneezing responses, we investigated whether an H1R antagonist, diphenhydramine, would be effective in attenuating these responses. Th2-transferred mice were intraperitoneally injected with diphenhydramine (500 μg), one day before the first OVA challenge and 60 min before each subsequent OVA challenge (Fig 6A). Diphenhydramine significantly suppressed the sneezing in Th2-transferred mice (Fig 6B), but did not affect the nasal infiltrations of Th2 cells and eosinophils (Fig 6C and 6D).

Recent studies have revealed that H4R antagonists diminish allergic inflammation in mouse models [6, 25]; therefore, we examined the efficacy of an H4R antagonist, JNJ7777120, in Th2- and macrophage-elicited rhinitis. Th2-transferred mice were intraperitoneally injected with JNJ7777120 (500 μg), one day before the first OVA challenge and 60 min before each subsequent OVA challenge (Fig 6A). Although JNJ7777120 did not affect the Th2- and macrophage-elicited sneezing responses (Fig 6E) and the nasal infiltrations of Th2 cells (Fig 6F), the nasal infiltrations of eosinophils were significantly decreased in the JNJ7777120-injected mice (Fig 6G). These data suggested that histamine is involved in the Th2- and macrophage-elicited sneezing responses through H1R signaling, whereas histamine caused the nasal infiltrations of eosinophils through H4R signaling.

## Discussion

The histamine produced by mast cells and basophils participates in allergic symptoms [1, 2]. However, in this study we found a novel histamine production mechanism involving the interaction of macrophages and Th2 cells. Our *in vitro* model suggested that the histamine production requires the direct interaction between macrophages and antigen-specific Th2 cells through the antigen. In addition, the *in vivo* model demonstrated that Th2 cells and macrophages cooperatively induce nasal allergic inflammation through histamine signaling.

Recent studies have indicated that macrophages, especially M2 macrophages, play important roles in allergic diseases [12]. Takamatsu *et al*. reported that LPS stimulation increases HDC expression and histamine production in BMDMs [15, 16]. Moreover, Murata *et al*. revealed that the granulocyte macrophage-colony stimulating factor (GM-CSF)-mediated increases in the histamine in monocytes/macrophages and the macrophage-derived histamine contribute to the pathogenesis of atherosclerosis [18]. We have shown that macrophages (both BMDMs and splenic macrophages) co-cultured with Th2 cells in the presence of antigen (OVApep) produce substantial amounts of histamine, but not in the absence of antigen. Furthermore, the source of the histamine in this co-culture model was macrophages (Figs 1 and 2). BMDMs produced histamine by interacting with naïve CD4 T cells through the antigen, however, activated Th2 cells had the stronger ability to induce histamine production from BMDMs (Fig 1A). We also revealed that BMDMs, but not splenic macrophages could produce histamine by interacting with antigen-specific Th1 cells through the antigen (S4 and S5 Figs). There seem to be different features between Th2 cells and Th1 cells *in vitro* model. The macrophages interacting with Th2 cells or Th1 cells may receive specific signals from them to increase histamine production. Although the precise cellular bases are unclear, this is a novel histamine production mechanism.

The *in vivo* model experiments demonstrated that the OVA challenge elicited sneezing responses in Th2-transferred mice (Fig 3B); however, these sneezing responses were markedly decreased in macrophage-depleted mice (Fig 4B) and *HDC*-deficient mice (Fig 5A). Based on these observations, we speculated that the sneezing responses elicited in Th2-transferred mice were induced in a macrophage/histamine-dependent manner. In our previous report, we showed that the OVA challenge induced sneezing responses in Th2-transferred mice in an IgE/mast cell/basophil-independent manner [13]. Since the Th2-transfer model used in this study (Fig 3A) closely resembled our previous Th2-transfer model (S8 Fig), the sneezing responses elicited by Th2 cells and macrophages were assumed to be induced in an IgE/mast cell/basophil-independent manner.

In addition to the *in vivo* observations, the *in vitro* data from the macrophage-Th2 cell co-culture model suggested that the histamine produced from macrophages interacting with Th2 cells might play important roles in the sneezing responses. Macrophages generally work as antigen-presenting cells to activate T cells *in vivo* [8, 9]. Therefore, it is likely that the macrophages and transferred-Th2 cells interacted with each other in the mouse nose. However, we could not directly prove that the histamine produced from macrophages induced the sneezing responses. Owing to the recent progress in genetic engineering, studies using conditional knockout mice, with the tissue- or cell-specific depletion of a target gene, have become more common. After establishing macrophage-specific *Hdc* gene-depleted mice, it will be possible to prove that the histamine derived from nasal macrophages is critical for the Th2- and macrophage-elicited sneezing responses. The most important issue is how histamine is related to the induction of sneezing responses. While the Th2 cell- and macrophage-elicited sneezing responses are considered to be induced in an IgE-mast cell/basophil-independent manner, the mechanisms remain unclear. Further studies are needed to reveal the precise mechanisms. Moreover, we need to investigate the relationship between histamine, macrophages, and Th1 cells *in vivo*, using our model of rhinitis. Because we aimed to elucidate the mechanisms of allergic rhinitis in this study, we analyzed Th2-transferred mice model. The *in vitro* data from the macrophage-Th1 cell co-culture model suggested that the histamine produced from macrophages interacting with Th1 cells might have important roles *in vivo*. There may be different features between Th2 cells and Th1 cells *in vivo* model, as well as *in vitro* model. In next study, we are going to analyze Th1-transferred mice model.

Although the antihistamine drugs used for AR treatment are targeted to H1R [3], many efforts have been made in the research and development of antiallergic drugs targeted to H4R over the past few years [4, 7], because H4R is expressed on T cells and eosinophils. Dunford *et al*. showed that H4R-deficient mice and mice treated with an H4R antagonist (JNJ7777120) exhibit decreased allergic lung inflammation [26]. Cowden *et al*. reported that JNJ7777120 significantly ameliorated allergen-induced T cell proliferation and cytokine production in a mouse model of asthma [27]. In our study, JNJ7777120 decreased nasal eosinophil infiltrations in the Th2-transfer model, without affecting the nasal activation of Th2 cells and sneezing responses (Fig 6E–6G). Ling *et al*. showed that histamine induces the upregulation of the adhesion molecules CD11b/CD18 (Mac-1) and CD54 (ICAM-1) on eosinophils, and mediates eosinophil chemotaxis. They also reported that these effects are mediated by H4R on eosinophils, and can be blocked by an H4R antagonist (JNJ7777120) [28]. Based on these findings, in our model, JNJ7777120 might have direct effects on eosinophils, leading to the reduction of nasal eosinophil infiltrations. We found that the H4R antagonist was a therapeutic candidate in AR. On the other hand, an H1R antagonist (diphenhydramine) administered one day before the nasal challenge suppressed the sneezing responses without affecting the nasal infiltrations of Th2 cells and eosinophils (Fig 6B–6D). However, the mechanism by which diphenhydramine suppresses sneezing responses, and the cells targeted by diphenhydramine remain enigmatic. Although more studies are necessary, our results suggest that Th2-elicited rhinitis is mediated by histamine through both H1R and H4R signaling.

Ohsawa *et al*. reported that a combined treatment with H1R and H4R antagonists has more significant therapeutic effects on chronic dermatitis in a mouse model, as compared to the single administration of each antagonist [29]. Mahapatra *et al*. also demonstrated that the combined treatment with H1R and H4R antagonists is superior to the treatment with single antagonists, in reducing the migration of antigen-specific Th2 cells to skin and Th2-dependent cytokine secretion [30]. Based on their observations, the co-administration of both H1R and H4R antagonists should be effective in patients with AR.

In summary, we have determined that macrophages produce histamine by interacting with Th2 cells *in vitro*, and Th2 cells and macrophages cooperatively induce nasal allergic inflammation through histamine signaling *in vivo*. Further studies must be conducted to reveal the precise mechanisms involved in the rhinitis elicited by Th2 cells and macrophages. We believe that our study will contribute to the development of novel therapeutic strategies toward nasal allergic disorders.

## Supporting information

S1 Fig The isolation of splenic macrophages. The purity of macrophages isolated from spleen (F4/80^+^CD11b^+^ cells in total isolation cells) were examined by FACS.
(TIF)

S2 Fig Th2 and Th1 differentiation. Th2 cells and Th1 cells were incubated with anti-CD3/CD28 Abs for 24h. Cytokine productions (IL-4 and IFN-γ) were measured by intracellular staining.
(TIF)

S3 Fig The expression of M2 macrophage markers. BMDMs were co-cultured with Th2 cells or naïve T cells in the presence of OVApep. The mRNA expressions of *Arg 1*and *Retnla* were measured in BMDMs. mRNA expressions were normalized to 18S rRNA levels. Data are shown by means±S.D. n = 3. **P<0.01, ****P<0.0001, N.S. not significant.
(TIF)

S4 Fig Macrophages produce histamine by interacting with antigen-specific Th1 cells. BMDMs and OVA-Th1 cells were co-cultured in the presence or absence of OVApep for 24 h, and then histamine production was measured in the culture supernatants. Co-culture of WT or *Hdc*^-/-^BMDMs with WT or *Hdc*^-/-^OVA-Th1 cells. Pooled data from 2 independent experiments are shown by means±S.D., n = 6. ***P<0.001, N.S. not significant.
(TIF)

S5 Fig Splenic macrophages did not produce histamine by interacting with antigen-specific Th1 cells through the antigen. Splenic macrophages (WT or *Hdc*^-/-^macrophages) and OVA-Th1 cells (WT or *Hdc*^-/-^OVA-Th1 cells) were co-cultured in the presence or absence of OVApep for 24 h, and then histamine production was measured in the culture supernatants. Data are shown by means±S.D., n = 3. N.S. not significant.
(TIF)

S6 Fig Gating strategy to analyze CD45+ hematopoietic cells and the frequencies of central memory T cells in spleen. (A) Representative flow cytometry plots of OVA-Th2 cells (CD4^+^DO11.10TCR^+^ cells in CD45^+^ cells) in spleen and nasal mucosa, eosinophils (CCR3^+^Siglec-F^+^ cells in CD45^+^ cells) in nasal mucosa, and central memory T cells (CD44^+^CD62L^+^ cells in CD45^+^CD4^+^DO11.10TCR^+^ cells) in spleen. (B) Frequencies of central memory T cells (CD44^+^CD62L^+^ cells in CD45^+^CD4^+^DO11.10TCR^+^ cells) in 5 weeks-Th2 cells and 2 days-Th2 cells were examined by FACS. Data are shown by means±S.D. n = 3. ***P<0.001.
(TIF)

S7 Fig Macrophage depletion in OVA-specific Th2 cell transfer model. (**A**, **B**) Frequencies of F4/80^+^CD11b^int^ macrophages (**A**) and CD11c^+^MHC-II^+^ dendritic cells (**B**) in total splenocytes were examined by FACS on day 4, immediately after the final challenge. Pooled data from 2 independent experiments are shown by means±S.D. n = 8. ****P<0.0001, N.S. not significant. (**C**) Representative flow cytometry plots of macrophages and dendritic cells.
(TIF)

S8 Fig Previous OVA-specific Th2 cells transfer model. Experimental schema. Mice were adoptively transferred with OVA-Th2 cells. After 2 days, mice were i.n. challenged.
(TIF)
