# Supplementary figures and images for: Th2 cells and macrophages cooperatively induce allergic inflammation through histamine signaling
Source: PLoS One. 2021 Mar 4;16(3):e0248158. doi: 10.1371/journal.pone.0248158 (PMC7932145; doi:10.1371/journal.pone.0248158)

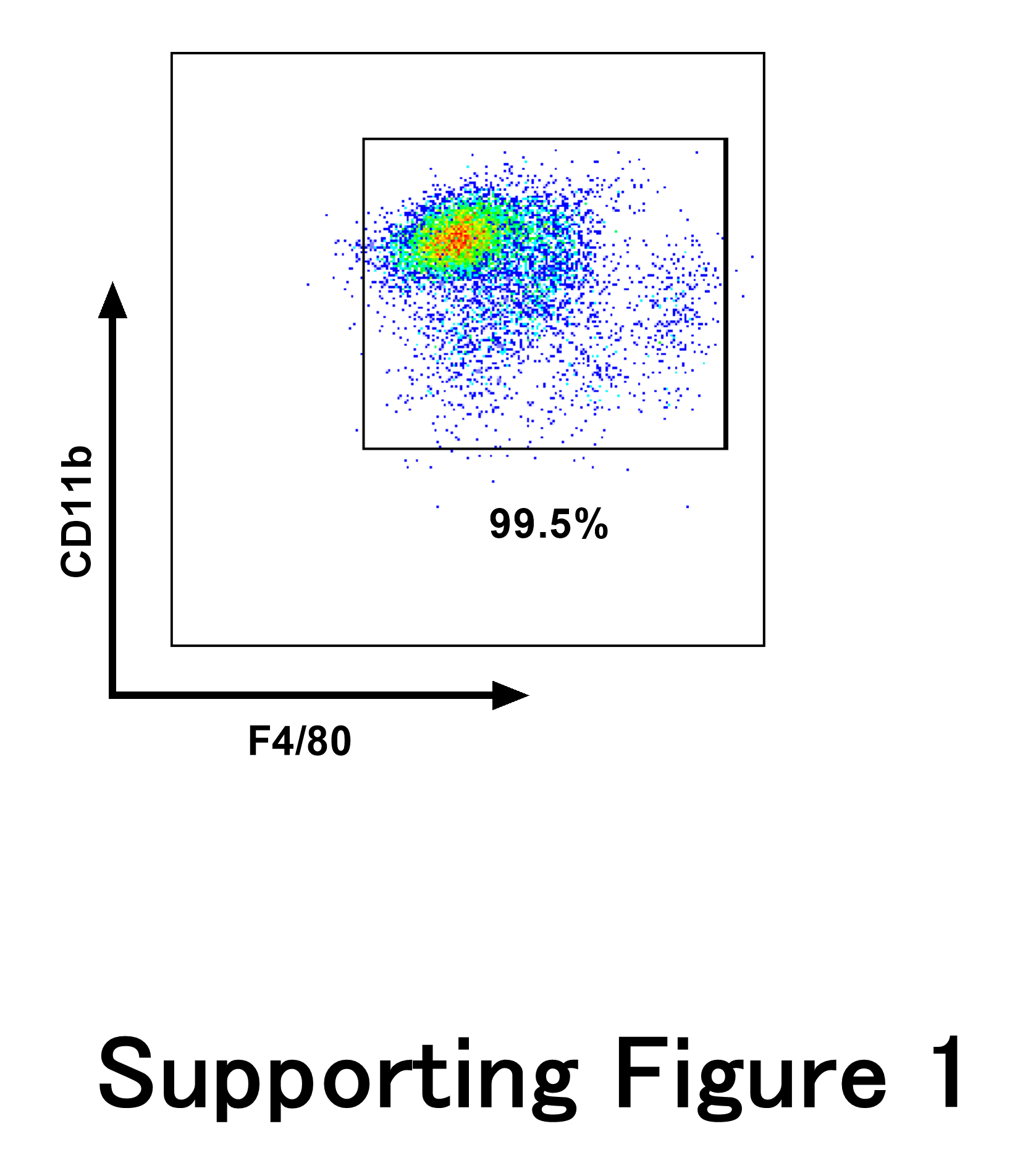

Supplement: S1 Fig — The purity of macrophages isolated from spleen (F4/80+CD11b+ cells in total isolation cells) were examined by FACS. (TIF) [file pone.0248158.s001.tif]

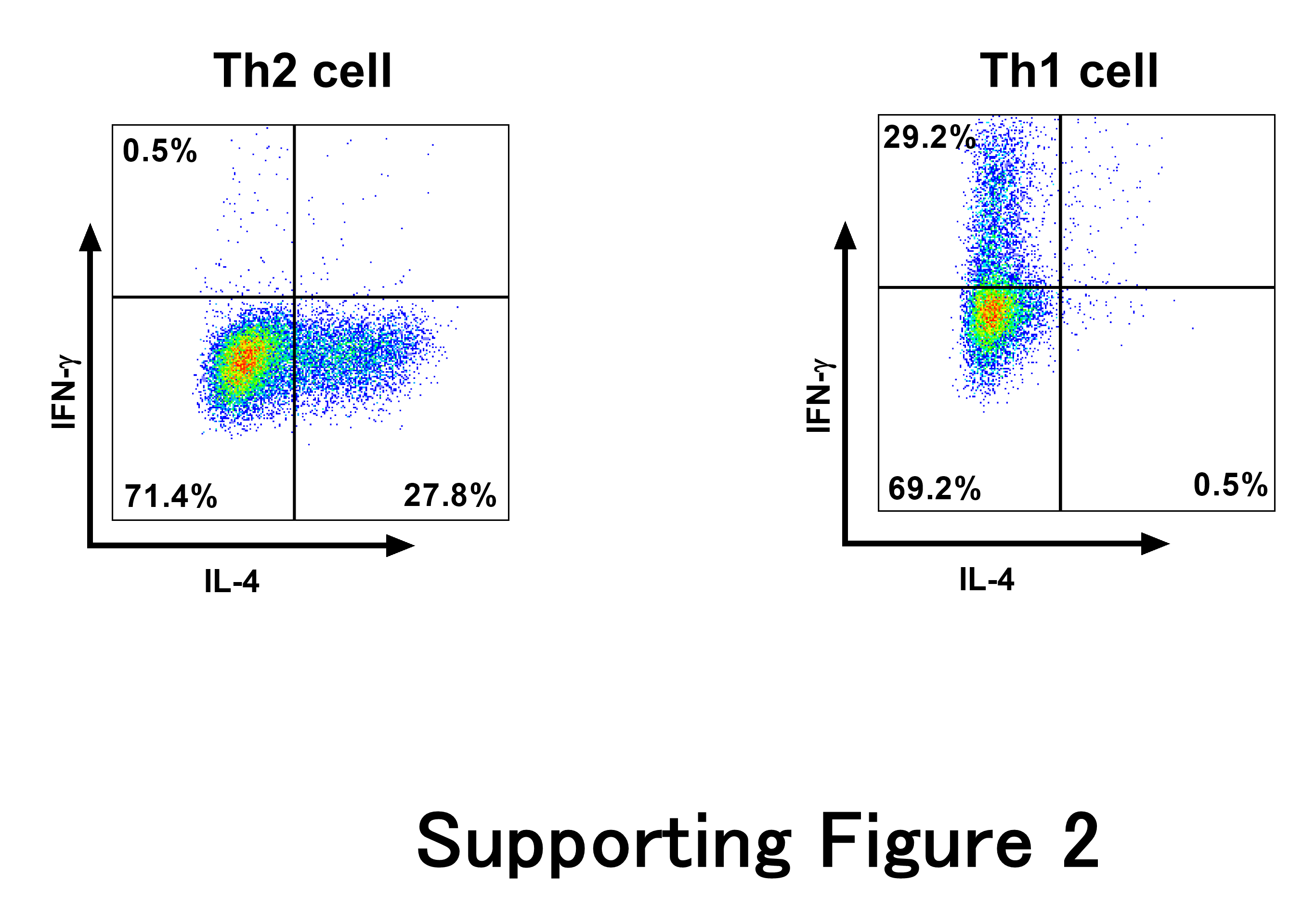

Supplement: S2 Fig — Th2 cells and Th1 cells were incubated with anti-CD3/CD28 Abs for 24h. Cytokine productions (IL-4 and IFN-γ) were measured by intracellular staining. (TIF) [file pone.0248158.s002.tif]

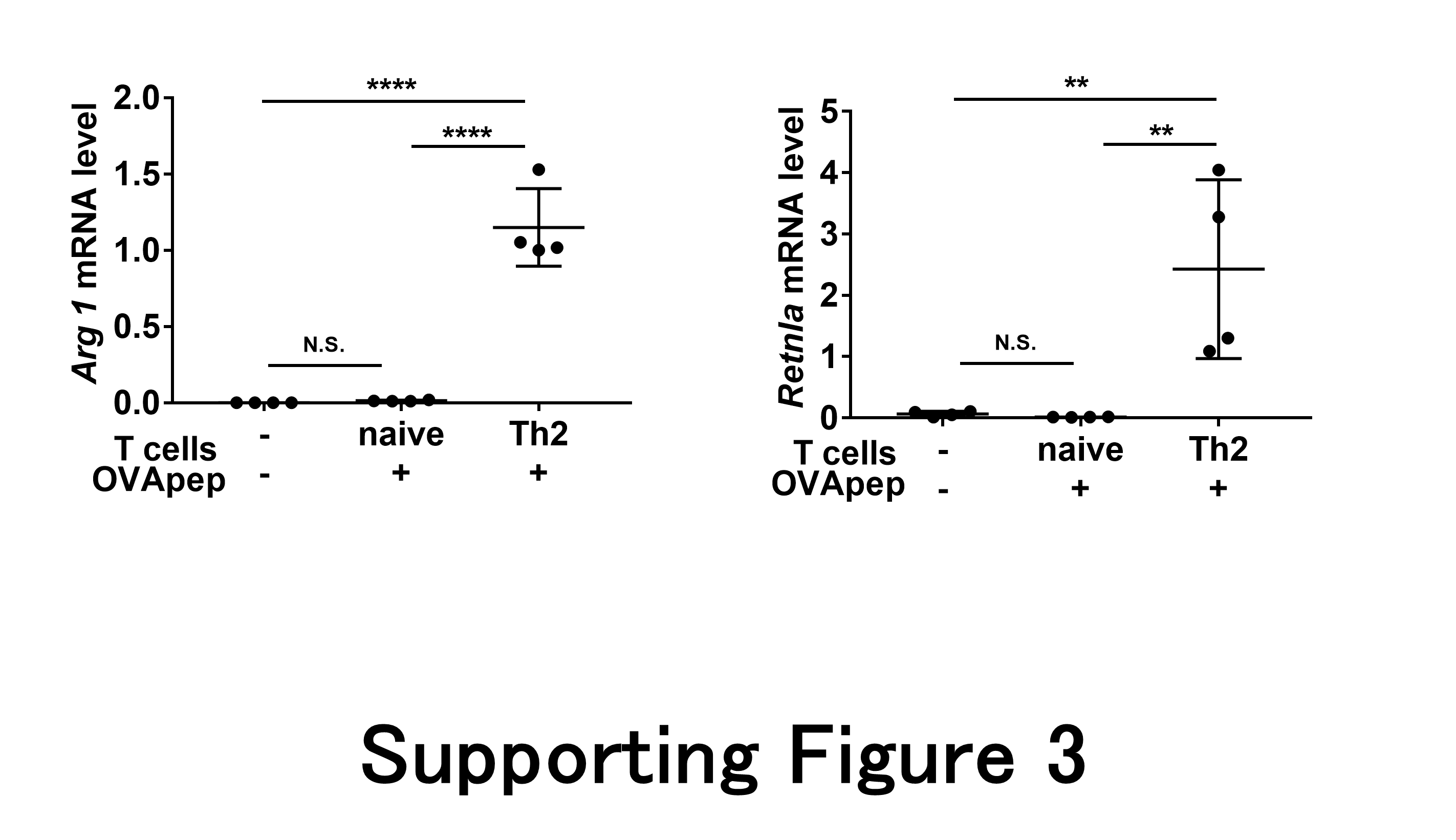

Supplement: S3 Fig — BMDMs were co-cultured with Th2 cells or naïve T cells in the presence of OVApep. The mRNA expressions of Arg 1and Retnla were measured in BMDMs. mRNA expressions were normalized to 18S rRNA levels. Data are shown by means±S.D. n = 3. **P<0.01, ****P<0.0001, N.S. not significant. (TIF) [file pone.0248158.s003.tif]

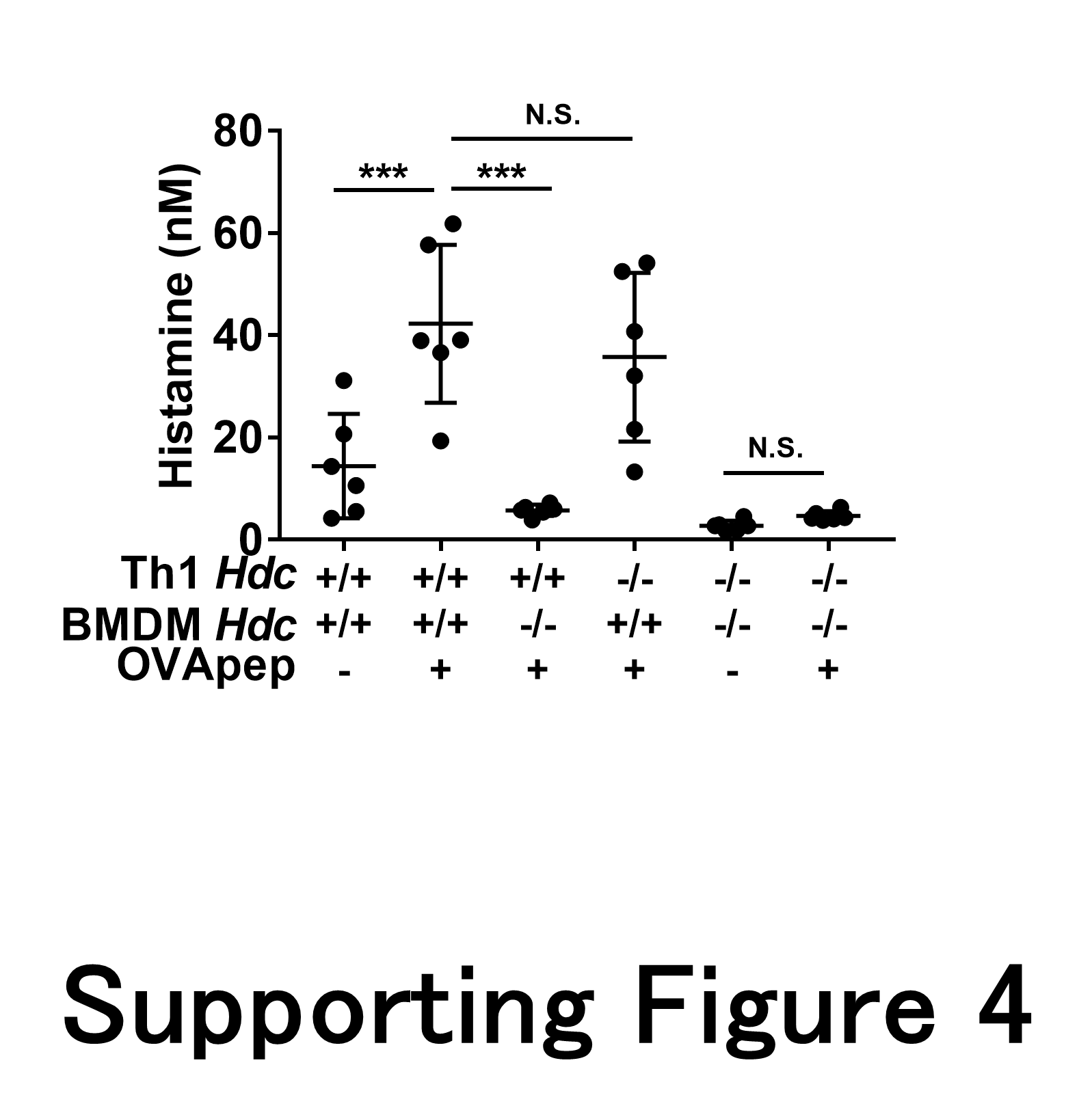

Supplement: S4 Fig — BMDMs and OVA-Th1 cells were co-cultured in the presence or absence of OVApep for 24 h, and then histamine production was measured in the culture supernatants. Co-culture of WT or Hdc-/-BMDMs with WT or Hdc-/-OVA-Th1 cells. Pooled data from 2 independent experiments are shown by means±S.D., n = 6. ***P<0.001, N.S. not significant. (TIF) [file pone.0248158.s004.tif]

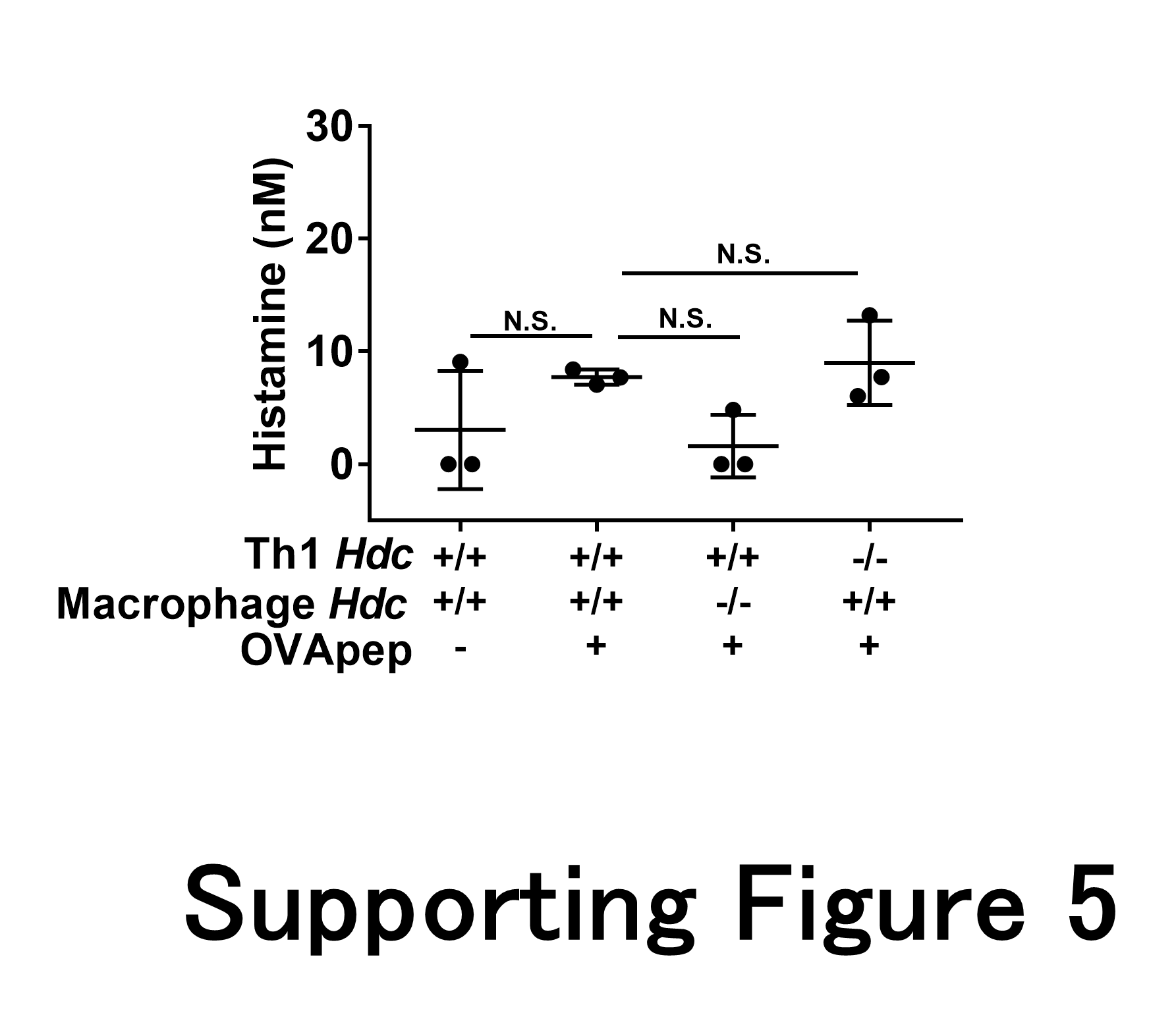

Supplement: S5 Fig — Splenic macrophages (WT or Hdc-/-macrophages) and OVA-Th1 cells (WT or Hdc-/-OVA-Th1 cells) were co-cultured in the presence or absence of OVApep for 24 h, and then histamine production was measured in the culture supernatants. Data are shown by means±S.D., n = 3. N.S. not significant. (TIF) [file pone.0248158.s005.tif]

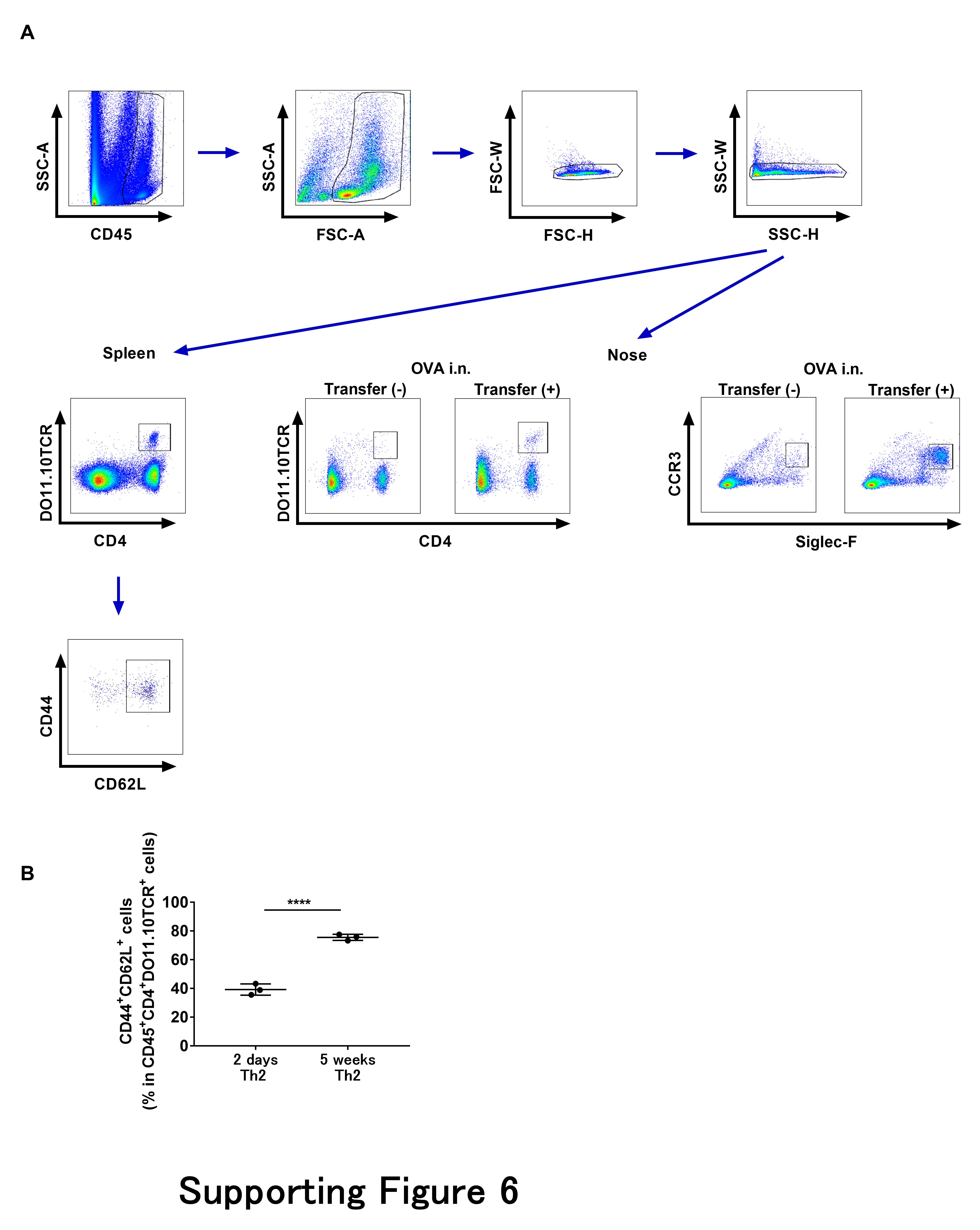

Supplement: S6 Fig — (A) Representative flow cytometry plots of OVA-Th2 cells (CD4+DO11.10TCR+ cells in CD45+ cells) in spleen and nasal mucosa, eosinophils (CCR3+Siglec-F+ cells in CD45+ cells) in nasal mucosa, and central memory T cells (CD44+CD62L+ cells in CD45+CD4+DO11.10TCR+ cells) in spleen. (B) Frequencies of central memory T cells (CD44+CD62L+ cells in CD45+CD4+DO11.10TCR+ cells) in 5 weeks-Th2 cells and 2 days-Th2 cells were examined by FACS. Data are shown by means±S.D. n = 3. ***P<0.001. (TIF) [file pone.0248158.s006.tif]

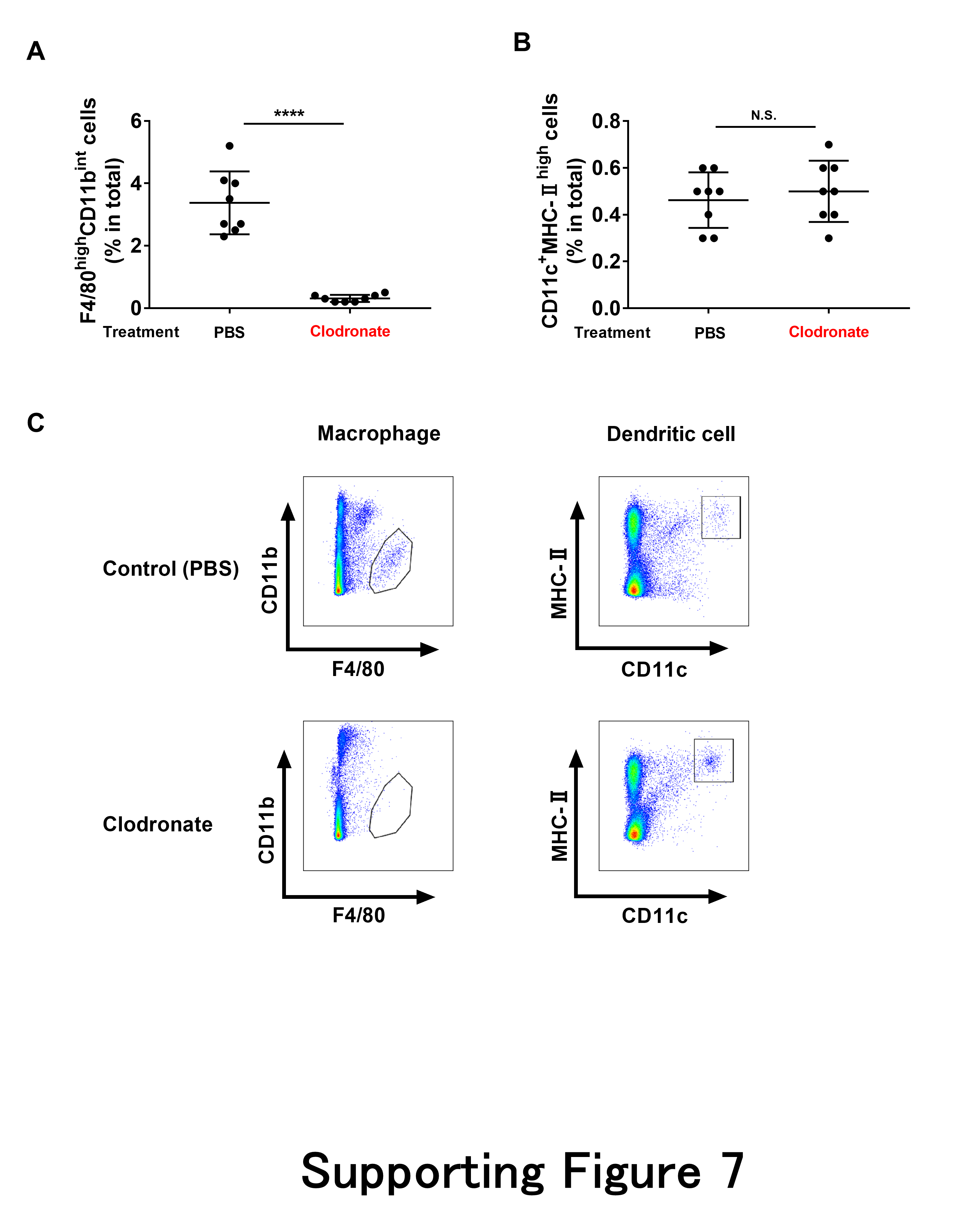

Supplement: S7 Fig — (A, B) Frequencies of F4/80+CD11bint macrophages (A) and CD11c+MHC-II+ dendritic cells (B) in total splenocytes were examined by FACS on day 4, immediately after the final challenge. Pooled data from 2 independent experiments are shown by means±S.D. n = 8. ****P<0.0001, N.S. not significant. (C) Representative flow cytometry plots of macrophages and dendritic cells. (TIF) [file pone.0248158.s007.tif]

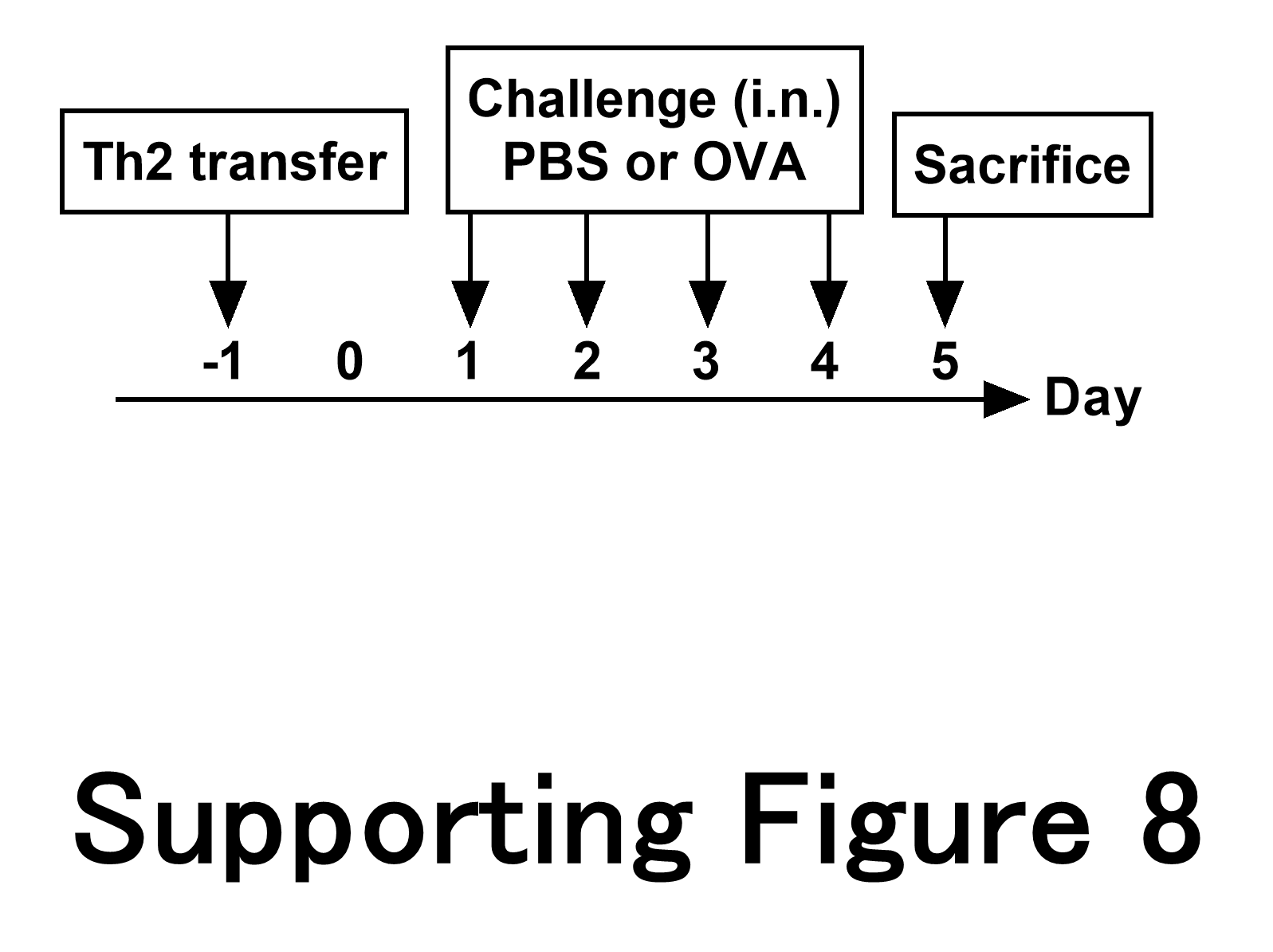

Supplement: S8 Fig — Experimental schema. Mice were adoptively transferred with OVA-Th2 cells. After 2 days, mice were i.n. challenged. (TIF) [file pone.0248158.s008.tif]
